# Simultaneous Quantification of Colistin A and B in Human Plasma Using a Small Volume with a High-Throughput LC-MS/MS Method

**DOI:** 10.3390/ph19060924

**Published:** 2026-06-12

**Authors:** Jimin Yoon, Won Gun Kwack, Kyung-Tae Lee, Eunseo Song, Hyeon Su Kim, Ki-Ho Park, Eun Kyoung Chung

**Affiliations:** 1 Department of Pharmacy, College of Pharmacy, Kyung Hee University, Seoul 02447, Republic of Korea; jm.yoon@khu.ac.kr (J.Y.); ktlee@khu.ac.kr (K.-T.L.); lucanus2@khu.ac.kr (H.S.K.); 2 Division of Pulmonary, Allergy and Critical Care Medicine, Kyung Hee University Hospital, Seoul 02447, Republic of Korea; wongunnim@naver.com; 3 Kyung Hee Drug Analysis Center, College of Pharmacy, Kyung Hee University, Seoul 02447, Republic of Korea; sssk2303@khu.ac.kr; 4 Department of Biomedical and Pharmaceutical Sciences, Graduate School, Kyung Hee University, Seoul 02447, Republic of Korea; 5 Department of Regulatory Science, Graduate School, Kyung Hee University, Seoul 02447, Republic of Korea; 6 Institutes of Regulatory Innovation Through Science (IRIS), Kyung Hee University, Seoul 02447, Republic of Korea; 7 Division of Infectious Diseases, Kyung Hee University School of Medicine, Seoul 02447, Republic of Korea; 8 Department of Pharmacy, Kyung Hee University Hospital at Gangdong, Seoul 05278, Republic of Korea

**Keywords:** colistin, colistin A, colistin B, LC–MS/MS, therapeutic drug monitoring, pharmacokinetics

## Abstract

**Background/Objectives:** Colistin is a complex polymyxin antibiotic with a narrow therapeutic window and significant interindividual pharmacokinetic variability, necessitating precise concentration monitoring. Current analytical methods often utilize colistin mixtures or require large sample volumes, potentially limiting the precision and resolution of individual component quantification. This study aimed to develop a sensitive and component-specific bioanalytical assay for the simultaneous quantification of colistin A and colistin B in human plasma. **Methods:** A liquid chromatography–tandem mass spectrometry (LC–MS/MS) method was developed using pure, component-specific reference standards to ensure rigorous independent quantification of each component. Analytes were efficiently extracted from a small volume of plasma (50 µL) using solid-phase extraction. Chromatographic separation was achieved on a C18 column with a total runtime of 4 min, and detection was performed using negative-ion multiple reaction monitoring (MRM). **Results:** Calibration curves showed excellent linearity over a range of 0.1–20 µg/mL for both colistin A and B (R^2^ > 0.99). The precision (%CV ≤ 8.8%) and accuracy (86.4–105.7%) for both components met the predefined regulatory criteria. This method was clinically validated using 60 plasma samples from 15 patients, demonstrating its applicability for capturing individual concentration–time profiles within the clinically relevant range (0.323–19.579 µg/mL for colistin A and 0.065–6.132 µg/mL for colistin B). **Conclusions:** This validated bioanalytical assay enables precise clinical pharmacokinetic assessments in a high-throughput workflow using a small plasma volume. Therefore, it serves as a practical tool for therapeutic drug monitoring (TDM)-guided dose optimization and further clinical investigations of colistin therapy.

## 1. Introduction

Colistin is a complex mixture of closely related compounds whose two principal constituents are colistin A (polymyxin E1) and colistin B (polymyxin E2); it also contains at least 30 minor polymyxins that share similar physicochemical properties [1,2]. It is administered intravenously as colistin methanesulfonate (CMS), an inactive prodrug that is hydrolyzed in vivo to the active components, colistin A and colistin B [3]. As a polymyxin antibiotic used to treat infections caused by multidrug-resistant Gram-negative bacteria [4,5], colistin has a narrow therapeutic window and substantial interindividual pharmacokinetic variability. Consequently, it is challenging to predict patient-specific plasma concentrations, and individualized dose optimization requires therapeutic drug monitoring (TDM) to manage clinical outcomes effectively [6,7,8].

Effective TDM requires a sensitive and robust bioanalytical method to quantify plasma drug concentrations accurately. Garonzik et al. (2011) demonstrated that the plasma exposure of formed colistin (the sum of colistin A and B concentrations) is highly variable among critically ill patients and is primarily determined by individual renal function [9]. The unpredictable variability necessitates precise monitoring to balance therapeutic efficacy against the significant risk of nephrotoxicity. Furthermore, the distinct antibacterial potencies and nephrotoxicity potentials of colistin A and B emphasize the importance of component-specific pharmacokinetic assessment for precise dosing.

Despite its clinical necessity, many LC-MS/MS assays rely on colistin sulfate mixtures, which often lack baseline chromatographic resolution between colistin A and B [10,11,12]. Gikas et al. (2013) [13] identified this as a critical limitation, emphasizing that in vivo A:B ratios can significantly deviate from the nominal ratio of administered bulk drug. Conventional assays measuring total colistin concentrations, often employed due to technical constraints, may not fully represent the specific pharmacological profile of each component [14,15].

To address these limitations, this study aimed to develop a sensitive and component-specific LC-MS/MS method for the simultaneous quantification of colistin A and B in human plasma. The proposed approach utilizes high-purity reference standards and a minimized sample volume of 50 µL to achieve high-throughput analysis without compromising analytical resolution. The method was rigorously validated according to regulatory guidelines to ensure its suitability for precise component-specific monitoring and clinical pharmacokinetic investigations [16,17].

## 2. Results

### 2.1. Development of LC-MS/MS Method

To establish an efficient, accurate, and reproducible LC-MS/MS assay for the respective quantification of colistin A and B, we screened several reversed-phase columns including Kinetex PS C18 (100 × 4.6 mm, 2.6 µm), Luna C8 (50 × 2.0 mm, 3 µm), HALO C18 (100 × 2.1 mm, 2.7 µm), and Capcell Pak C8 (50 × 2.0 mm, 5 µm). Tested mobile phases were as follows: 0.1% formic acid in water and acetonitrile, 10 mM ammonium acetate (pH 3.2), 20 mM ammonium formate (pH 2.5), acetonitrile, tetrahydrofuran, 60% methanol in deionized water (DW), and 60% isopropanol in DW, using both isocratic and gradient programs. Kinetex PS C18 (100 × 4.6 mm, 2.6 µm) was ultimately selected for its superior peak shape, minimal tailing, clean baseline, and consistent retention that afforded baseline separation of colistin A and B with low carryover. The finalized chromatographic condition, together with optimized source/MRM settings, enabled efficient quantification of colistin A and B from small plasma volumes of 50 µL within a high-throughput workflow across the validated range.

### 2.2. Validation of the LC-MS/MS Method

#### 2.2.1. Selectivity and Sensitivity

**Figure 1:**
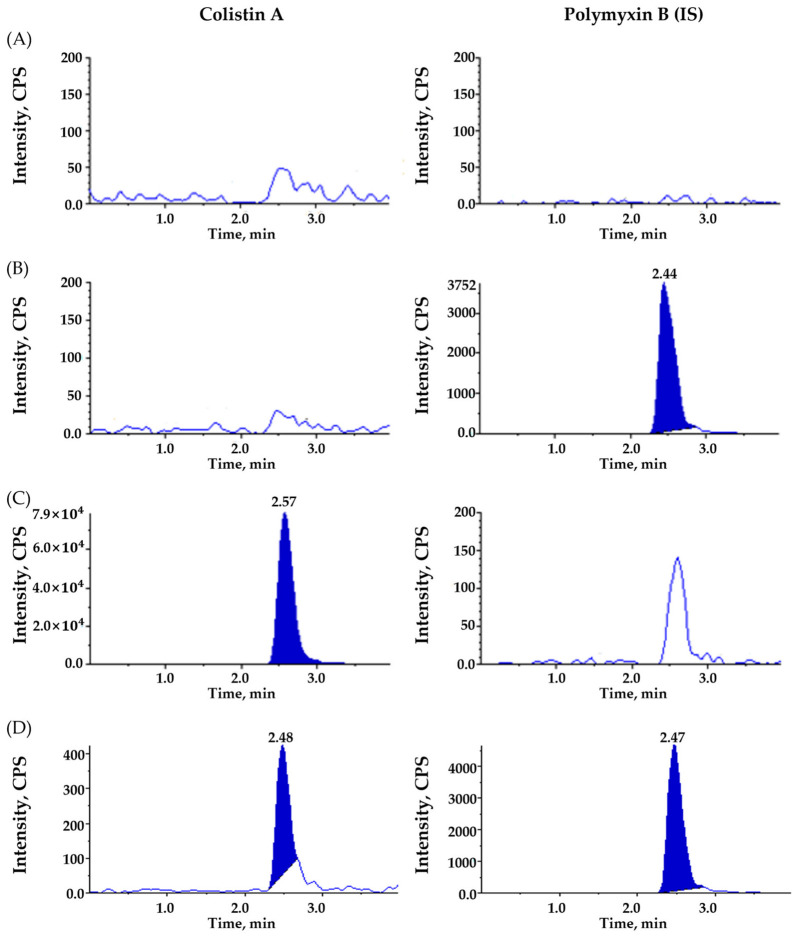
Representative chromatograms for colistin A in human plasma: (**A**) double blank; (**B**) blank spiked with polymyxin B, as the internal standard (IS, 1 µg/mL); (**C**) blank spiked with colistin A (upper limit of quantification, 20 µg/mL); (**D**) blank spiked with colistin A (lower limit of quantification, 0.1 µg/mL) and polymyxin B (IS, 1 µg/mL). Panels on the left side are for colistin A, and those on the right side are for IS.

**Figure 2:**
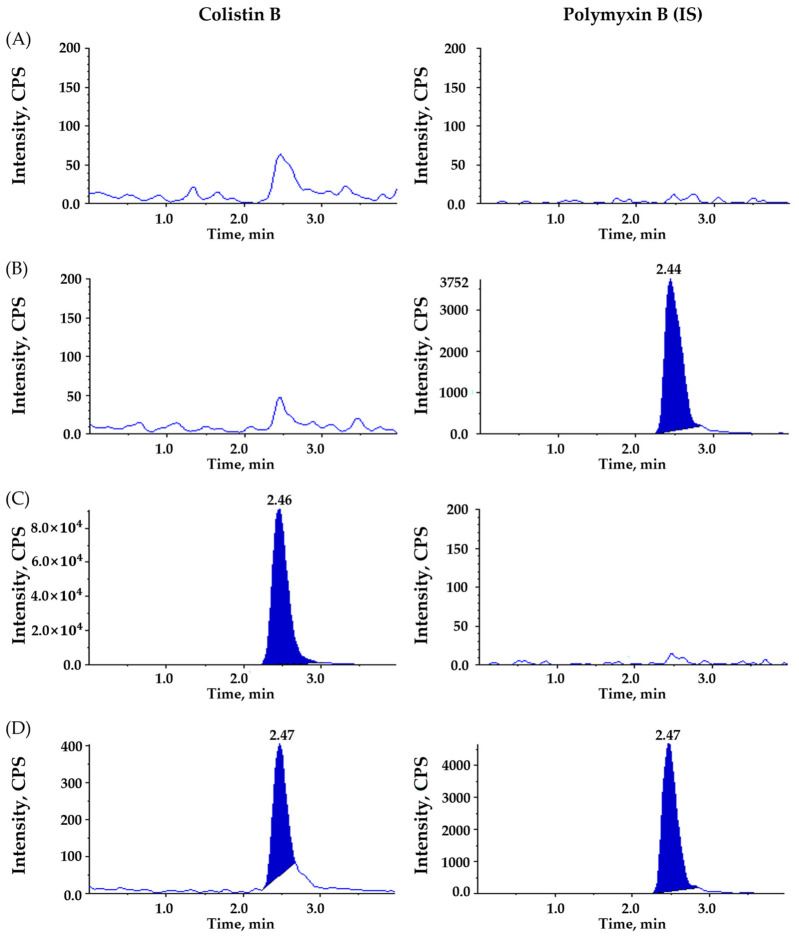
Representative chromatograms for colistin B in human plasma: (**A**) double blank; (**B**) blank spiked with polymyxin B, as the internal standard (IS, 1 µg/mL); (**C**) blank spiked with colistin B (upper limit of quantification, 20 µg/mL); (**D**) blank spiked with colistin B (lower limit of quantification, 0.1 µg/mL) and polymyxin B (IS, 1 µg/mL). Panels on the left side are for colistin B, and those on the right side are for IS.

No interfering peaks were observed at the retention times of colistin A, colistin B, or the IS. The signal-to-noise (S/N) ratio was greater than 10 at the LLOQ (Figure 1 and Figure 2).

LLOQ was validated using seven replicate samples. All S/N ratios were above 10, and CVs for peak area ratios were 6.5% for colistin A and 11.3% for colistin B, within the ±20% acceptance range.

#### 2.2.2. Linearity

Linearity was confirmed across seven concentration levels (0.1, 0.5, 1, 2, 5, 10, 20 µg/mL). All calibration runs showed coefficients of determination (R^2^) > 0.99 and Pearson’s correlation coefficient (r) > 0.9951. Representative linear equations are presented below, including the mean ± standard deviation (SD) of the slope and y-intercept:

Colistin A: y = 0.825 (±0.025)x − 0.00193 (±0.0045)

Colistin B: y = 0.932 (±0.011)x − 0.00742 (±0.0084)

#### 2.2.3. Precision and Accuracy

Accuracy was estimated to range from 94.1% to 100.4% (between runs) and 95.0% to 101.5% (within runs) for colistin A. At the LLOQ, accuracy was 94.1% (between runs) and ranged from 91.8% to 95.6% (within runs). For colistin B, accuracy ranged from 86.4% to 105.7% (between runs) and 87.9% to 102.9% (within runs), with the corresponding values at the LLOQ of 105.7% and from 99.4% to 114.4%, respectively. All values were within acceptable criteria (Table 1). Precision was evaluated at four QC levels. For colistin A, between-run precision ranged from 2.9% to 6.6% and within-run from 1.3 to 8.2%. At the LLOQ, precision was estimated to be 6.6% (between runs) and 3.0% to 8.2% (within runs). For colistin B, between-run precision ranged from 4.0% to 8.8%, and within-run ranged from 2.5% to 5.7%, with the corresponding values at the LLOQ of 8.8% (between runs) and from 5.7% to 7.9% (within runs). All results were within acceptable limits (Table 1).

#### 2.2.4. Recovery, Matrix Effect, and Carryover

Recovery and matrix effect were evaluated at three concentrations (Table 2). The IS-normalized recovery ranged from 92.8% to 103.3% for colistin A, and from 21.2% to 23.8% for colistin B. The CVs of recovery were 4.3–15.3% for colistin A and 5.2–22.6% for colistin B. Matrix effects ranged from 20.2% to 42.0% for colistin A and from 76.7% to 143.8% for colistin B. The CVs of matrix effect were 8.4–30.0% for colistin A, 4.5–23.7% for colistin B. Despite these variations, no baseline abnormalities were observed in the chromatograms.

Carry-over was evaluated by comparing two DB injections before and after ULOQ injections. No carry-over was detected for colistin A, colistin B, or IS.

#### 2.2.5. Stability

Stability test results satisfied the pre-specified acceptance criteria under all assessed conditions (Table 3). Following three freeze–thaw cycles, colistin A and colistin B retained 97.2–107.3% and 89.0–104.9% of their initial concentrations, respectively. Short-term stability for 7 h was confirmed at room temperature (99.7–103.4% for colistin A and 87.7–101.7% for colistin B), at 4 °C (100.6–102.7% and 86.1–103.4%), and at −70 °C (96.8–104.4% and 88.8–101.8%). Autosampler stability for 24 h at 4 °C yielded stability data in the ranges of 97.0–104.5% for colistin A and 88.4–104.4% for colistin B.

#### 2.2.6. Dilution Integrity

Dilution integrity was validated by diluting 2-fold QC samples (i.e., 0.6, 15, 32 µg/mL) of colistin A with blank plasma and injecting three replicates. Colistin A concentrations remained within the range of 99.3–110.4%, confirming integrity for diluted samples.

### 2.3. Application to a Clinical Pharmacokinetic Study

**Figure 3:**
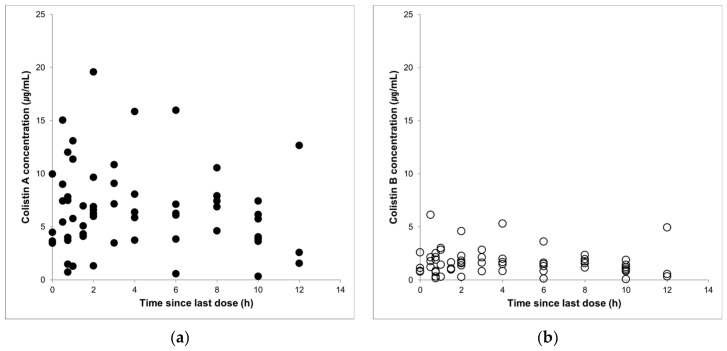
Observed (**a**) colistin A and (**b**) colistin B concentrations in plasma samples at multiple time points from 15 patients receiving intravenous colistin methanesulfonate (i.e., CMS).

The clinical applicability of the developed LC-MS/MS assay was evaluated by quantifying colistin A and colistin B in plasma samples from 15 adult patients receiving colistin therapy for MDR infections. As shown in Figure 3, the assay demonstrated adequate sensitivity, with the established LLOQ encompassing the clinically observed concentration ranges for both components. The wide vertical dispersion of these data points highlights the substantial interindividual pharmacokinetic variability of colistin, particularly in critically ill patients with varying degrees of renal impairment. In patient samples, colistin A and colistin B were quantified at 0.323–19.579 µg/mL and 0.065–6.132 µg/mL, respectively. All measured values fell within the established calibration range, with the exception of a single colistin B concentration (0.065 µg/mL) that was below the LLOQ (0.1 µg/mL).

## 3. Discussion

This study successfully developed and validated a component-specific LC-MS/MS assay for the simultaneous quantification of colistin A and B using a minimized plasma volume. These findings carry substantial implications for both bioanalytical validation and individualized TDM.

This method utilizes a minimal sample volume of 50 µL, representing a significant reduction compared to conventional LC-MS/MS protocols that typically consume 100–1000 µL of plasma [13,18,19,20,21,22,23]. These minimal volume requirements are particularly beneficial for vulnerable patient populations, such as elderly or pediatric groups, where frequent and repeated blood sampling is often restricted by physiological limitations. Furthermore, the simultaneous quantification of both components optimizes laboratory throughput and enhances clinical workflow efficiency. Such practical benefits facilitate the rapid reporting of colistin levels, supporting timely dose adjustments in acute care settings where managing patients with fluctuating renal function is critical.

While conventional pharmacokinetic models have utilized total colistin concentrations due to analytical constraints, this approach may fail to account for the distinct pharmacological profiles of colistin A and B [10,11,12]. Specifically, Gikas et al. (2013) noted that methods lacking chromatographic separation may present concerns for the evaluation of low drug levels [13]. By addressing these technical limitations, our method provides a higher-resolution tool to investigate the variability in colistin exposure described by Garonzik et al. (2011) [9]. Given that colistin A and B exhibit independent in vivo kinetic pathways [14,15], absolute quantification using individual pure standards enables a more detailed interpretation of patient plasma profiles than the summation of components. The observed concentration ranges (0.323–19.579 μg/mL for colistin A and 0.065–6.132 μg/mL for colistin B) include the commonly targeted therapeutic range of approximately 2 μg/mL for total colistin. Differentiated monitoring of these components allows for a more precise assessment of individual exposure, which is critical for managing patients with fluctuating renal clearance. The higher concentrations observed in several patients were consistent with their severely impaired renal function (eGFR < 20 mL/min/1.73 m^2^), emphasizing the critical role of this method in TDM-guided dose optimization to mitigate the risk of nephrotoxicity.

To ensure the absolute reliability of such clinical monitoring, the bioanalytical validation parameters were rigorously evaluated. The IS-normalized recovery for colistin A reached 103.3% at high QC level. This slight excess is attributed to non-specific adsorption (NSA) in the post-extraction matrix and the shielding effect in the pre-extraction plasma matrix [24,25]. In the post-extraction phase, the purified reconstitution solvent can promote minor NSA of colistin A to the container surfaces, which decreases the peak response [25]. Conversely, endogenous proteins in the pre-extraction plasma shield the analyte from such surface adsorption during initial sample handling. This differential surface loss between the two states can cause the calculated recovery to marginally pass 100%, a phenomenon well-documented in peptide bioanalysis [24].

Similarly, although colistin B exhibited a low recovery range of 21.2–23.8%, the extraction process was consistent across three concentration levels. Regulatory guidelines state that extraction recovery does not need to be near 100%, provided that the process is precise and reproducible [16,17]. Polymyxin B, serving as a structural analogue internal standard, effectively tracked and compensated for this extraction behavior, ensuring accurate and reliable quantification.

Furthermore, the analytical sensitivity of the assay also meets clinical requirements. Although a single clinical sample concentration was below the LLOQ (0.065 μg/mL), this exceptionally low trough concentration is well below the generally accepted target average steady-state concentration of 2 μg/mL for total colistin [6,9]. Because this sub-therapeutic concentration is clinically negligible, the lack of precise quantification does not compromise the clinical utility of the assay or dosage decision-making. Consequently, these findings demonstrate the clinical robustness and utility of the assay for monitoring patients across diverse physiologic conditions.

Overall, the performance and efficacy of this method support its use as a practical and sensitive tool for clinical monitoring. Furthermore, by independently quantifying colistin A and colistin B components with distinct antibacterial potencies and varying nephrotoxicity risks, this study demonstrates the clinical utility of component–specific TDM. Such differentiated quantification enables precise dose adjustments and supports the transition toward individualized colistin therapy through comprehensive pharmacokinetic profiling. Although the current study is limited by a sample size of 15 patients due to the practical challenges in recruiting critically ill patients, these results successfully demonstrate the clinical applicability of the method in a real-world setting. Further large-scale investigations are necessary to apply this component-specific approach to a large number of clinical samples and to establish definitive population pharmacokinetic models.

## 4. Materials and Methods

### 4.1. Chemicals and Standards

Colistin A (purity 90%), colistin B (purity 90%), and polymyxin B (purity 95%; internal standard, IS) were obtained from Toronto Research Chemicals. All standards were stored at −20 °C. All chemicals used were of analytical grade.

Human plasma used for validation was collected from Bestian Hospital (Chungcheongbuk-do, Republic of Korea), immediately heparinized, and stored at −70 °C. Blank plasma was used for preparing calibration and quality control (QC) samples.

Stock solutions of colistin A and colistin B were prepared at 1000 µg/mL in deionized water. Polymyxin B stock solution was prepared in 100% methanol at 1000 µg/mL. Working solutions for colistin A and B (1–200 µg/mL) were prepared in 0.1% formic acid in deionized water, and the IS working solution (1 µg/mL) was prepared in 50% methanol in water. All stock solutions were stored at −20 °C.

Calibration standards were prepared by spiking blank plasma to yield final concentrations of 0.1–20 µg/mL for both colistin A and B. QC samples were prepared at 0.1 µg/mL (lower limit of quantification [LLOQ]), 0.3 µg/mL (low), 7.5 µg/mL (mid), and 16 µg/mL (high). Additional QC samples for validation tests (recovery, matrix effect, etc.) were prepared at 0.3, 7.5, and 16 µg/mL. All validation samples were freshly prepared on the day of analysis.

### 4.2. Instrumentation and Chromatographic Conditions

Chromatographic analysis was performed using an Agilent 1200 Series HPLC system (Agilent Technologies, Santa Clara, CA, USA) and an API 4000 triple quadrupole mass spectrometer (Applied Biosystems, Framingham, MA, USA). Separation was achieved using a Phenomenex Kinetex PS C18 2.6 µm, 100 × 4.6 mm column (Phenomenex, Torrance, CA, USA).

The mobile phase consisted of mobile phase A (0.5% acetic acid in water) and mobile phase B (acetonitrile) in a 70:30 (*v*/*v*) ratio, filtered and ultrasonically degassed. The flow rate was 300 µL/min, with a total runtime of 4 min.

**Figure 4:**
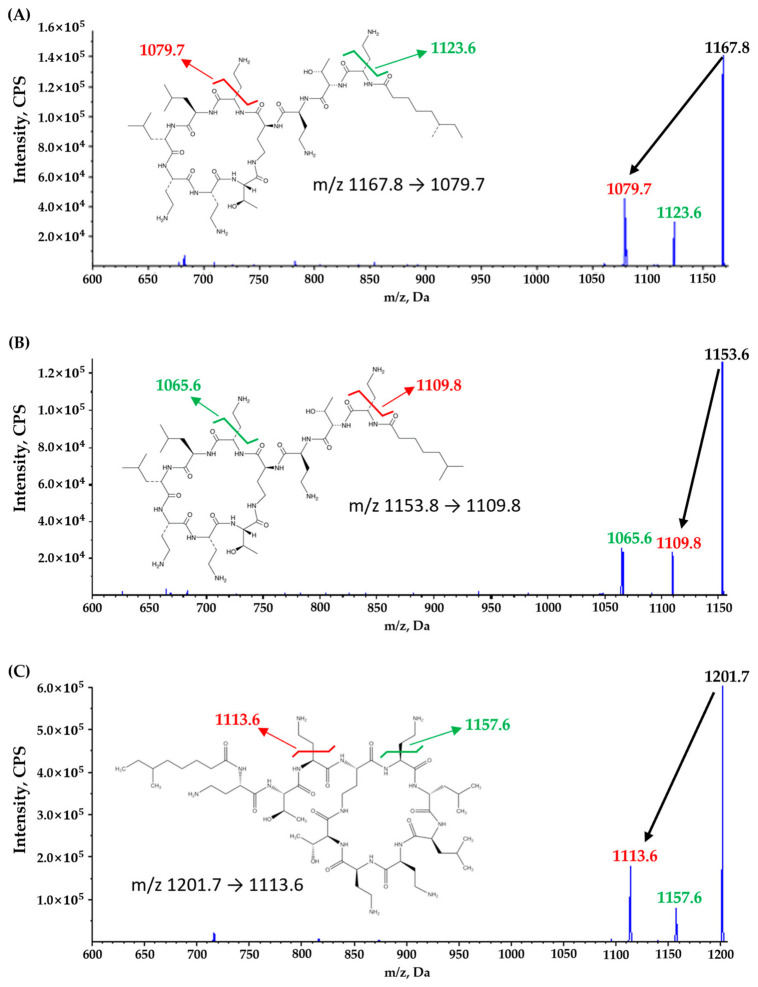
Product ion mass spectra and fragmentation patterns of (**A**) colistin A, (**B**) colistin B, and (**C**) polymyxin B (internal standard, IS). Black arrows indicate the specific *m*/*z* transitions used for the following multiple-reaction-monitoring (MRM)-based quantification: 1167.8 → 1079.7 for (**A**), 1153.8 → 1109.8 for (**B**), and 1201.7 → 1113.6 for (**C**). Red and green lines denote the putative fragmentation sites leading to the formation of the primary product ions and alternative fragment ions.

Detection was performed in negative electrospray ionization (ESI−) mode using multiple reaction monitoring (MRM). Transitions were as follows: colistin A (*m*/*z* 1167.8 > 1079.7), colistin B (*m*/*z* 1153.8 > 1109.8), and IS (*m*/*z* 1201.7 > 1113.6) (Figure 4). The optimized MS parameters are summarized in Table 4. Analyst 1.6.2 software was used for data analysis.

### 4.3. Sample Preparation

Forty microliters of blank plasma was spiked with 5 µL of either colistin A or B working solution in addition to 5 µL of IS to make a final volume of 50 µL. Concentrations were set at 0.1–20 µg/mL for calibration and 0.1, 0.3, 7.5, 16 µg/mL for QC.

Samples were processed using solid-phase extraction. Strata-X cartridges (Phenomenex, Torrance, CA, USA) were conditioned with methanol and ammonium hydroxide. Samples, already spiked with IS, were loaded onto the cartridges, washed with 1.5 mL of 100% methanol once, and eluted using 2.5% formic acid in methanol. Eluates were evaporated under nitrogen gas at 40 °C and reconstituted with 300 µL of a mixture of acetonitrile and 0.1% formic acid in water (50:50, *v*/*v*). After centrifugation at 20,800× *g*, 150 µL of supernatant was transferred to vials, and 15 µL was injected into the LC-MS/MS system.

### 4.4. Method Validation

Validation included selectivity, sensitivity (LLOQ), linearity, accuracy, precision, recovery, matrix effect, carry-over, stability, and dilution integrity. All acceptance criteria were established according to the guidelines of United States Food and Drug Administration (FDA) and the Ministry of Food and Drug Safety in Korea (MFDS) [16,17].

Selectivity was evaluated using six blank plasma sources. Double blank (DB), zero blank (ZB), blank spiked with either colistin A or B, and blank sample spiked with IS as well as colistin A or B at LLOQ were analyzed. Analyte responses at the retention times were not considered as signal if they were below 20% of the LLOQ response; for the IS, responses below 5% of the reference IS response were not considered as signal.

Linearity was assessed for both colistin A and B within the concentration range of 0.1–20 µg/mL (0.1, 0.5, 1, 2, 5, 10, and 20 µg/mL), which was selected to encompass the TDM range of colistin in clinical human plasma. Calibration curves were constructed with linear regression describing the relationship between the nominal concentration (x) and the peak area ratio (analyte to IS; y) using 1/x^2^ as the weighting factor.

Accuracy was measured as deviation from nominal concentrations. Mean values had to be within ±15% (±20% for LLOQ). Precision was assessed at four standard concentrations, defining acceptable precision as within- and between-run CVs ≤ 15% (≤20% for LLOQ).

Recovery was determined as IS-normalized recovery. It was calculated by comparing peak area ratios of analyte to IS between pre- and post-extraction samples at three standard concentrations [26]. Matrix effect was assessed at three QC levels by comparing post-extraction and pure standard peak areas. CVs ≤ 15% were considered acceptable. The calculation formulas were as follows.

$$\mathrm{Recovery}\;(\mathrm{IS-normalized},\;\%)=\frac{{\left({\mathrm{Area}}_{\mathrm{analyte}}/{\mathrm{Area}}_{\mathrm{IS}}\right)}_{\mathrm{pre-extraction}}}{{\left({\mathrm{Area}}_{\mathrm{analyte}}/{\mathrm{Area}}_{\mathrm{IS}}\right)}_{\mathrm{post-extraction}}}\times 100$$

$$\mathrm{Matrix}\;\mathrm{Effect}\;(\%)=\frac{{\mathrm{Area}}_{\mathrm{post-extraction}}}{{\mathrm{Area}}_{\mathrm{pure}\;\mathrm{standard}}}\times 100$$

Carry-over was tested using DB and upper limit of quantification (ULOQ) samples. Acceptance criteria were <20% of LLOQ signal for analytes and <5% for IS.

Stability was assessed under freeze–thaw cycling, short-term (room temperature [RT], 4 °C, −70 °C), and autosampler conditions. Coefficients of variation under all conditions within ±15% were considered acceptable.

The assessment of dilution integrity was performed to ensure analytical reliability for samples with limited volumes or those with concentrations exceeding the ULOQ. For the plasma matrix, the dilution integrity was evaluated using colistin A as a representative analyte; spiked samples were prepared in three replicates and diluted two-fold with blank human plasma to yield final concentrations of 0.3, 7.5, and 16 µg/mL. The resulting accuracy and precision were considered acceptable if they remained within ±15% of the nominal values. This verification confirms that the dilution process does not compromise the validity of the quantification, ensuring robust performance for clinical sample analysis across an extended concentration range.

### 4.5. Clinical Application

The validated LC-MS/MS method was applied to determine the plasma concentrations of colistin A and colistin B in 15 adult patients requiring colistin therapy for infections caused by multidrug-resistant (MDR) Gram-negative bacteria. The dosage of colistin was administered at the clinical discretion of the treating physician based on each patient’s clinical condition. The study included critically ill patients with significant renal impairment, specifically those with an estimated glomerular filtration rate (eGFR) below 20 mL/min/1.73 m^2^ as calculated by the MDRD equation.

Blood sampling was performed at least two days after initiation of colistin treatment. For each patient, four blood samples were collected via a central venous catheter. Sampling time points were randomly selected from a protocol-defined schedule ranging from pre-dose to subsequent dose. All samples were collected in heparinized tubes and immediately centrifuged at 20,800× *g* for 10 min at 4 °C. The plasma samples were then stored at −70 °C until analysis. This study was conducted in accordance with ethical standards and was approved by the Institutional Review Board (IRB) of Kyung Hee University Hospital in Seoul (IRB no.: 2018-10-036).

## 5. Conclusions

In this study, we established and validated a high-throughput LC-MS/MS assay that enables precise, component-specific quantification of colistin A and B using a minimal plasma volume of 50 μL and individual reference standards. This method improves analytical reliability and enables each component to be monitored separately, providing a robust tool to characterize the distinct antibacterial potencies and varying nephrotoxicity risks of colistin A and B. Consequently, this method is expected to be a useful tool for TDM-based dose optimization and clinical pharmacokinetic studies, supporting individualized colistin therapy in clinical practice.

## Figures and Tables

**Table 1:** Between-run and within-run precision and accuracy of colistin A and B in human plasma by LC-MS/MS.

Nominal Concentration (µg/mL)	Colistin A			Colistin B		
	Predicted Concentration (Mean ± SD, µg/mL)	Precision (CV, %) ^a^	Accuracy (%) ^b^	Predicted Concentration (Mean ± SD, µg/mL)	Precision (CV, %) ^a^	Accuracy (%) ^b^
Inter-assay precision and accuracy (*n* = 3 runs of sample replicates)						
0.1	0.094 ± 0.006	6.6	94.1	0.106 ± 0.009	8.8	105.7
0.3	0.293 ± 0.019	6.4	97.6	0.302 ± 0.012	4.0	100.7
7.5	7.532 ± 0.222	2.9	100.4	7.568 ± 0.303	4.0	100.9
16	15.792 ± 0.696	4.4	98.7	13.816 ± 0.587	4.2	86.4
Intra-assay precision and accuracy (*n* = 5 sample replicates)						
0.1	0.095 ± 0.008	8.2	95.0	0.099 ± 0.006	5.7	99.4
0.3	0.296 ± 0.010	3.4	98.5	0.309 ± 0.013	4.1	102.9
7.5	7.470 ± 0.097	1.3	99.6	7.583 ± 0.203	2.7	101.1
16	16.232 ± 0.977	6.0	101.5	14.059 ± 0.356	2.5	87.9

^a^ Precision (CV, %) = (standard deviation of the predicted concentrations/nominal concentration) × 100. ^b^ Accuracy (%) = (predicted concentration/nominal concentration) × 100. CV: coefficient of variation; SD: standard deviation.

**Table 2:** Recovery (IS-normalized) and matrix effect of colistin A and colistin B in human plasma using solid phase extraction (*n* = 6, different blank plasma).

Nominal Concentration (µg/mL)	Recovery (Mean ± SD, %)	Matrix Effect (Mean ± SD, %)
Colistin A		
0.3	92.8 ± 14.2	42.0 ± 12.6
7.5	97.8 ± 10.2	21.8 ± 5.3
16	103.3 ± 4.4	20.2 ± 1.7
Colistin B		
0.3	23.8 ± 3.4	143.8 ± 34.1
7.5	23.5 ± 5.3	76.7 ± 11.7
16	21.2 ± 1.1	83.0 ± 3.7

IS: internal standard; SD: standard deviation.

**Table 3:** Stability data (mean ± standard deviation, %) for colistin A and colistin B in human plasma.

Stability Condition	Nominal Concentration (µg/mL)					
	Colistin A			Colistin B		
	0.3	7.5	16	0.3	7.5	16
Freeze–thaw stability (three cycles)	107.3 ± 1.4	101.2 ± 0.6	97.2 ± 6.9	104.9 ± 3.8	101.1 ± 2.3	89.0 ± 5.2
7 h, Room temperature	99.7 ± 4.9	100.8 ± 2.0	103.4 ± 1.3	101.7 ± 4.6	100.2 ± 2.6	87.7 ± 3.0
7 h, 4 °C	102.7 ± 5.4	102.2 ± 2.7	100.6 ± 2.4	103.4 ± 5.3	98.8 ± 2.5	86.1 ± 2.6
7 h, −70 °C	96.8 ± 2.4	102.1 ± 1.6	104.4 ± 2.2	101.1 ± 4.3	101.8 ± 2.8	88.8 ± 1.8
24 h, Autosampler (4 °C)	97.0 ± 1.8	104.5 ± 1.7	103.5 ± 3.4	101.4 ± 3.3	104.4 ± 3.0	88.4 ± 4.1

**Table 4:** Mass spectrometer conditions for colistin A, colistin B, and polymyxin B (IS).

Compound	DP (V)	EP (V)	CE (V)	CXP (V)	DT (s)
Colistin A	−185	−10	−34	−37	0.15
Colistin B	−170	−10	−34	−35	0.15
Polymyxin B	−190	−10	−58	−51	0.15

DP: declustering potential; EP: entrance potential; CE: collision energy; CXP: cell exit potential; DT: dwell time.

## Data Availability

The original contributions presented in this study are included in the article. Further inquiries can be directed to the corresponding authors.
